# Bayesian Multimodel Inference for Geostatistical Regression Models

**DOI:** 10.1371/journal.pone.0025677

**Published:** 2011-11-10

**Authors:** Devin S. Johnson, Jennifer A. Hoeting

**Affiliations:** 1 National Oceanic and Atmospheric Administration (NOAA) National Marine Mammal Laboratory, Seattle, Washington, United States of America; 2 Department of Statistics, Colorado State University, Fort Collins, Colorado, United States of America; University of Bristol, United Kingdom

## Abstract

The problem of simultaneous covariate selection and parameter inference for spatial regression models is considered. Previous research has shown that failure to take spatial correlation into account can influence the outcome of standard model selection methods. A Markov chain Monte Carlo (MCMC) method is investigated for the calculation of parameter estimates and posterior model probabilities for spatial regression models. The method can accommodate normal and non-normal response data and a large number of covariates. Thus the method is very flexible and can be used to fit spatial linear models, spatial linear mixed models, and spatial generalized linear mixed models (GLMMs). The Bayesian MCMC method also allows *a priori* unequal weighting of covariates, which is not possible with many model selection methods such as Akaike's information criterion (AIC). The proposed method is demonstrated on two data sets. The first is the whiptail lizard data set which has been previously analyzed by other researchers investigating model selection methods. Our results confirmed the previous analysis suggesting that sandy soil and ant abundance were strongly associated with lizard abundance. The second data set concerned pollution tolerant fish abundance in relation to several environmental factors. Results indicate that abundance is positively related to Strahler stream order and a habitat quality index. Abundance is negatively related to percent watershed disturbance.

## Introduction

Ecologists and other environmental scientists often consider a large number of plausible regression models in an effort to explain ecological relationships between several covariates and a response variable. Model selection procedures, such as Akaike's information criterion (AIC), are routinely employed to help researchers decide upon an appropriate model to describe the environmental system [1].

In addition to the increase in the use of model selection methods, advancing technology has led to the routine use of global positioning systems (GPS) to collect spatially referenced data. The increase in spatial data collection has led environmental scientists to recognize that there may be substantial spatial correlation present in their data. As a result spatial correlation models have become more popular in recent years. Here we investigate a model selection method for geostatistical regression models. In addition to estimating regression coefficients, a geostatistical regression model involves fitting a spatial correlation function to the regression errors. This function allows correlation between observations to decrease as separation in space increases. These models are traditionally termed universal kriging models [2]. The kriging terminology, however, refers to spatial prediction and ecologists are often more interested in inference concerning the covariate portion of the model. Therefore, the term geostatistical regression is used for a spatially correlated regression analysis.

In most regression model selection methods, spatial correlation is ignored. This can lead to erroneous inference of the importance of some covariates in explaining variation in the response variable [3]. Hoeting et al. [4] explore use of AIC for geostatistical regression models. Thompson [5] considers a Bayesian approach to geostatistical regression selection and model averaging predictions using integral approximations to obtain the necessary model weights.

In this paper a Bayesian selection procedure for geostatistical regression models was investigated using a Markov chain Monte Carlo (MCMC) approach. Bayesian and MCMC methods are becoming increasingly popular in the ecological literature [6]–[9]. Chapter 7 in Givens and Hoeting [10] provides an introduction to MCMC procedures. Green [11] proposed reversible-jump MCMC (RJMCMC) as a method for traversing model space as well as parameter space. This allows one to make Bayesian inference on the model set in an MCMC setting.

The RJMCMC method presented in this paper has several advantages. Many covariates can be examined through a stochastic model search. The prior weighting of the coefficients is another benefit over methods such as AIC. Certain covariates can be given more or less weight *a priori*. Methods such as AIC selection weight all covariates equally. The RJMCMC approach has one other major advantage over AIC and Bayesian closed form approximations, it is directly extendable to spatial generalized linear mixed models (GLMMs). This implies the RJMCMC approach can be an all purpose tool for geostatistical regression inference for Gaussian and non-Gaussian data. In order to demonstrate the method in either scenario, we present two example analyses. The first examines the previously analyzed whiptail lizard data set. We chose this data set as a way to assess the accuracy of the method. The whiptail data set has been analyzed several times [3]–[5] with spatial regression models each time the same general model has been chosen. Thus, we analyzed it with our method to confirm the results. The second analysis examined fish abundance data using a Poisson spatial GLMM to illustrate the extension to non-normal data.

## Methods

### Geostatistical regression models

The geostatistical model [2] is a commonly used model for spatially referenced data in a continuous spatial domain. A general model that allows both normal and non-normal response data is presented so that the method can be applied in a variety of ecological studies. In order to account for spatial correlation in normal and non-normal data [12] propose using a spatial GLMM. The spatial GLMM approach uses a traditional GLM with a spatially correlated random effect in order to obtain spatial correlation in the observed data.

Let 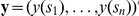 be a set of spatially referenced measurable observations, where 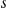 denotes a location in a spatial domain 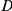 (usually a two dimensional region). In a spatial GLMM the response variables, 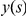 are assumed to be independent given an underlying Gaussian spatial process 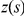 and the vector of covariates 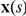. That is to say 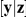 is distributed according to 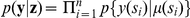, where 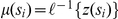 is the conditional mean of 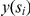 and 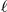 is a link function.

The geostatistical process 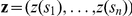 is assumed to have the normal distribution 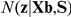, where 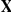 is a matrix of covariates with each row associated with site 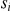, 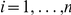. The spatial covariance matrix is defined by the spatial covariance function of the form
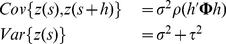
(1)where 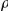 is an isotropic correlation function, 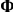 is a 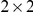 positive definite matrix that allows for geometric anisotropy [2], 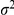 is the sill parameter, and 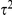 is the nugget parameter. The correlation function 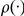 may take many forms. Typical choices are the exponential, Matern, or spherical correlation functions [13], [14].

The joint density of the observations 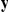 and the spatial process 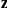 is given by 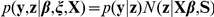, where 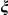 is a vector of the five spatial covariance parameters 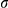, 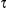 and the unique elements of 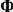. Hence, the likelihood for the geostatistical regression model is given by 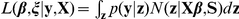. There is only one common observation model for which this likelihood can be obtained in closed form, the normal distribution. In this case, the resulting likelihood is the multivariate normal 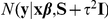 distribution.

### Bayesian inference

As the model is presented in the previous section there are significant difficulties in making Bayesian inference. Previous MCMC analysis of spatial data has noted a high posterior correlation between 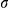 and 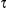 making MCMC samplers slow to converge [15]. Therefore, we use the alternate parameterization 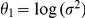 and 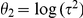. This significantly reduced correlation in the MCMC samples the examples considered herein.

In addition, because 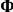 must remain positive definite (i.e., 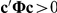 for all vectors 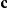) there are several awkward constraints on the entries of 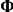. To remove the constraints, we reparameterized by factoring the anisotropy parameters as 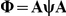, where 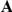 is a diagonal matrix with positive elements and 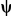 is a positive definite correlation matrix. Let 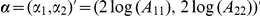. Any 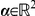 is valid allowing a large range of possible priors. Due to the fact that 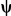 is a 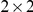 correlation matrix, it has but one parameter 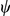 that represents the angle of anisotropy. The valid range of 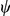 in that case is (−1, 1). The elements of the original anisotropy matrix 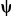 can be rewritten as functions of 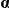 and 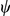, so 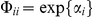 for 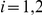, and 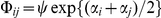 for 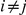. The resulting collection of spatial correlation and variance parameters is given by 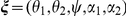.

### Model uncertainty

Bayesian inference for the spatial regression model is based on the posterior distribution 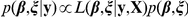, where 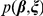 is the parameter prior distribution. Desired quantities for summarization of the density are usually in the form of expected values, for example posterior means, variances, and percentiles or credible intervals. The posterior density is intractable; therefore, these quantities can be approximated from an MCMC sample.

The Bayesian approach to model uncertainty assumes that the model itself, like the parameter values, is an unknown entity. Therefore, the joint posterior distribution of the parameters and the model is of interest. The joint posterior for model 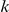 is given by
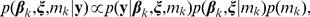
(2)where 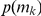 is the prior probability of the 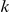
th model and 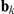 is a vector of coefficients for the covariates in model 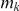. A classic model prior for regression analysis is derived by treating inclusion of the 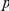 coefficients as a series of independent Bernoulli trials with probability 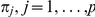 [16]. The result is the following prior
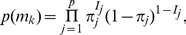
(3)where 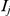 is an indicator that covariate 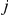 is included in the regression model. This prior includes the uniform prior 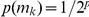; obtained by setting 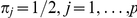.

In most model selection problems the object of inference is not the joint model-parameter posterior, it is the marginal posterior distribution of the model set. This marginal distribution is the posterior model probability (PMP) for model 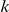, given by
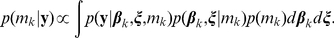
(4)The PMP is almost always unobtainable in closed form. Typically, the model with the largest PMP is selected. Alternatively, one may not want to select a specific model, but use all of the models, appropriately weighted by their PMPs, in an ensemble fashion. Hoeting et al. [17] provide a detailed description of this type of inference termed Bayesian Model Averaging (BMA).

This paper will use both BMA and maximum PMP to make inference concerning the importance of each covariate in explaining an ecological or environmental response. The maximum PMP model provides information on important covariates. Another quantity, the posterior inclusion probability (PIP), is also useful in regression settings. The PIP for the 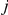
th covariate is defined
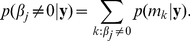
(5)This is the model averaged posterior probability of inclusion of the 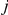
th covariate. The PIP for each covariate provides a measure of importance of each covariate to the response.

### Reversible-Jump MCMC

Unlike ordinary regression, closed forms of the PMPs and PIPs cannot be obtained for spatial regression models. Green [11] proposed the RJMCMC method for sampling from the joint space of the parameters and model. The key difference between MCMC and RJMCMC is that the parameter space changes as the chain moves between models. This makes the algorithm construction more complex. Sample averages can then be used to approximate expected values of model and parameter functions, such as PMPs and PIPs.

A major challenge of the general RJMCMC method is that a double proposal is necessary to move to a different model. First, an appropriate model must be proposed, followed by an acceptable proposal for the parameters of the model. If either of these two proposals is inefficient then the chain will fail to mix well and a large number of iterations will be necessary to do posterior model inference. This can make computations very slow in the spatial regression case because the 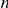 by 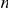 covariance matrix 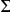 that must be inverted at every MCMC iteration.

In order to avoid long RJMCMC runs with spatial regression models an efficient proposal scheme is necessary. Godsill [18] suggested a general proposal method for model classes where some of the parameters are shared among each model. In the spatial regression case, the spatial parameters 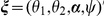 are common to all of the models, whereas 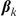 differs for each model. If the conditional posterior distribution of the model given the shared parameters is available than a more efficient Partial Analytic RJMCMC (PARJ) algorithm can be constructed. Using the basic idea of Godsill, a PARJ chain was constructed for spatial regression model moves in the following manner. For a current state 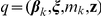,

1. Update 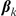, 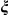 using the full conditional distributions as in a standard Gibbs sampler (details in File S1).
2. Update 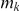 with PARJ as follows
  - Propose model move to 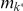 with probability 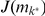,
  - Propose 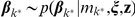,
  - Set 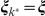 and 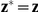,
  - Accept 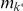 with probability
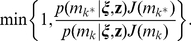
(6)
3. Update the entire vector 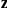 using a Langevin-Hastings proposal [19] (See File S1 for details).

Upon examination of (6), one can see there is no need to draw 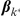 proposal values due to the fact that the conditional distribution
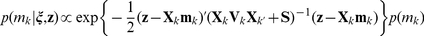
(7)is available in closed form when 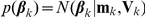. Details of the calculation of 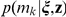 are given in File S1. In the present examples we use a discrete random walk for 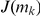. First, a covariate is chosen with probability 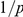, if it is already in 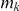, it is removed in 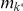 if it is not in 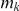 it is added. Thus, 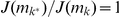. Table 1 summarizes the steps in the PARJ algorithm. In the following sections we demonstrate the method with the analysis of two separate data sets.

**Table 1:** Steps in PARJ algorithm for geostatistical regression models.

Step	Update type	Proposal distribution^a^
1. Update 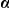	Metropolis	Gaussian
2. Update 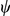	Metropolis	Truncated uniform (−1, 1)
3. Update 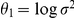	Metropolis	Gaussian
4. Update 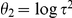	Metropolis	Gaussian
5. Update 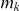	PARJ	Discrete random walk
6. Update 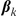	Gibbs	Gaussian^b^
7^c^. Update 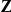	Langevin-Hastings	Gaussian

The pollution intolerant fish abundance data were analyzed using the following model and parameter updating scheme. All Metropolis proposal distributions are random walks centered on the current parameter value.

a Metropolis proposal distributions are centered on the current parameter value.

b Full conditional distribution.

c Step 7 is only necessary for spatial GLMMs.

### Example 1: Analysis of Whiptail Lizard Abundance

The proposed model selection methodology was applied to the previously analyzed whiptail lizard data set in order the compare the RJMCMC method to other more traditional methods. The data were initially analyzed using a stepwise procedure with a spatial correlation correction [3]. The data were subsequently analyzed using a BIC approximation to the PMP [5] and finally using AIC [4]. Each of these analyses demonstrate the danger of ignoring spatial correlation when selecting covariates. A larger model is often selected to account for the ignored correlation. We used this data set to determine whether the method and proposed RJMCMC algorithm performed as expected.

The data set is composed of abundance data of the Orange-throated whiptail lizard in Southern California. At 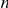 = 149 locations where lizards were observed the average number of lizards trapped during a week long trapping period was recorded. The response variable analyzed is the log transformed value 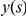 = ln(average no. trapped at location 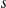).

Several covariates were collected to investigate which environmental conditions explain lizard abundance. The original set of environmental covariates contained 37 variables. After initial screening [5] six covariates remained which held potential to explain lizard abundance: Crematogaster ant abundance (3 levels - low, medium, high), log percent sandy soils, elevation, a bare rock indicator, percent cover, and log percent chaparral plants. Using indicators for ant level 1 and 2, there are 128 possible models. All 6 covariates were normalized to have mean zero and variance 1.

The PARJ approach was used to select covariates with spatial correlation present. Here, the full spatial model is used with nugget and anisotropy. The exponential function 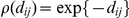, where 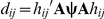 was used to model spatial correlation. None of the previous analyses included anisotropy effects. In addition, a model without spatial correlation was analyzed as well to determine any effects that might occur when correlation is ignored in the selection process.

Following [5] and [20], the mean and variance of the 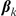 normal prior was chosen to be 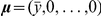, where 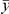 the sample mean of 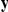. The prior covariance was chosen to be 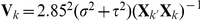. Priors for the variance parameters 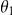 and 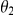 where chosen to be 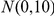. The sample variance 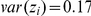, therefore, a prior variance of 10 on the log variance parameters provided adequate coverage over the set of realistic values of the partial sill and nugget. For each range parameter 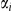, a 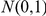 prior distribution was used. Automatically choosing extremely large variances for the range priors can put an unrealistic amount of mass on ranges well beyond maximum observed distances, which can adversely affect results. A variance of 1 distributed 90% of the prior mass of the correlation range (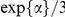) less than the maximum observed distance. A uniform distribution can be used for a noninformative prior on 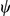, however, a triangular distribution over (−1, 1) and centered on 0 produced a more stable MCMC analysis. This was due to the fact that 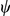 values near 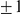 tended to cause inconsistencies in the covariance structure.

The PARJ chain was run for 100,000 iterations following a sufficient burn-in period. See Table 2 for a list of tuning parameters used in the RJMCMC within model moves. We did not thin the chain as that is primarily a tool to relieve storage issues for high dimensional parameters. The number of parameters in this analysis (and the following example) is not that great as to cause computer storage problems. However, if one would like to monitor model averaged predicted values of the response variable at many sites, thinning may become necessary. In order to judge whether the run length was acceptable, the Heidleberger-Welch diagnostic test [21] was performed on the parameters common to all models. Visual inspection also confirmed that this was an acceptable chain length. See File S2 for more details and figures. The analysis was performed using the R statistical package. Code is available in the File S3.

**Table 2:** Proposal distributions for RJMCMC within model moves of each analysis.

Parameter	Whiptail lizard	Fish abundance
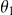	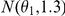	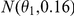
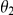	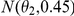	-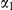	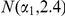	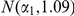
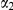	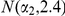	-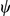	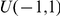	-

Each proposal was a random walk, therefore it is centered on the previous parameter value in the RJMCMC iteration.

### Example 2: Abundance of Pollution Tolerant Fish

In this section the PARJ approach is demonstrated on a data set of fish counts for several geographically referenced sites. A Poisson model with spatially correlated random effects model is adopted for the observed counts. Model selection analysis is performed to determine which of several environmental factors contribute to overall abundance of pollution-intolerant fish.

In 1994 and 1995 numerous stream sites in the Mid-Atlantic region of the Unites States (Maryland, Pennsylvania, Virginia, West Virginia) were visited as part of the U.S. Environmental Protection Agency's EMAP water quality monitoring program. Several stream characteristics were measured to assess water quality. At 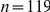 sites fish were sampled and classified according to their pollution tolerance. When assessing water quality the abundance of pollution intolerant fish at a site is often a good index.

The emphasis of the analysis is the effects of pollution and stream quality variables, however, there are several natural factors which might also influence abundance. The natural covariates include Strahler stream order (ORDER), elevation (ELEV), and watershed area (WSA). The remaining covariates can be modified by human use and, therefore, were considered potential stream quality variables. Stream quality variables included: road density in the watershed (RD) (No./area), percentage of watershed classified as disturbed by human activity (DISTOT), an index of fish habitat quality at the stream site (HAB), concentration of dissolved oxygen in the stream at the sampling site (DO), % areal fish cover at the sampling site (XFC), and percent sand in stream bed substrate (PCT). Strahler order is a measure of how far a particular section of stream is from its headwater sources. For example, headwater streams have order 1. Two order 1 streams merge to form an order 2 stream, etc. Thus ORDER is an index of stream size and relative location in the watershed. The covariates in this analysis have vastly different scales, therefore, to increase MCMC mixing, the covariates were standardized to have mean 0 and variance 1.

It is believed *a priori* that the natural variables should be included in the final model, but, this is not known with certainty. Therefore, the natural variables will be more heavily weighted in the prior model probabilities. For the natural variables prior inclusion probabilities were set to 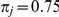, while for the remaining disturbance variables 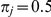. This *a priori* weighting illustrates one of the advantages of the Bayesian approach to selection. In addition, the data were also analyzed with a flat model prior (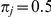 for all 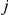) to examine sensitivity of this prior weighting.

A Poisson distribution with the canonical log link function is chosen for the abundance model. So, the likelihood is given as
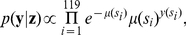
(8)where 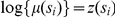 and 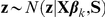.

Priors for 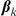 and 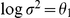 were chosen to be 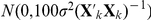 and 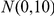 respectively. Here we chose a flatter prior for 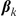 than in the previous example in order to have minimal prior influence on the coefficients. This was the first examination of this data in a model selection context, whereas we chose a more informative prior in the whiptail analysis so the results were comparable to previous analyses. As in the previous example, a variance of 10 for the 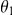 prior placed significant mass beyond the scale of the measured data, therefore, it was sufficiently vague, but still proper. An isotropic exponential spatial correlation model was used for covariate selection after initial analysis of the full model suggested no significant anisotropy or nugget effect. The Poisson observation model essentially takes the place of the nugget measurement error variability in the Gaussian process. We assumed that the range parameter 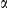 followed a 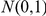 distribution. This prior placed more than 90% of the prior mass below the maximum observed range to maintain stable inference.

The PARJ chain was run for 300,000 iterations after a sufficient initial burn-in. Table 2 presents the proposal distributions used for within model moves. In the Langevin-Hastings updates of 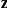, a value of 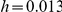 was used providing an acceptance rate of approximately 50%. New models were proposed via the random walk approach of randomly selecting a covariate for addition or deletion from the current model. See File S2 for the convergence diagnostic analysis. Code for analysis within the R statistical language is available in File S3.

## Results

### Example 1: Analysis of Whiptail Lizard Abundance

The top five models in PMP are given in Table 3. The PARJ algorithm visited 114 out of 128 possible models. The top model included Ant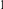 and log percent sandy soil. With only 17.8% posterior model mass, however, there is considerable uncertainty in the best model. Under the independence assumption, the top PMP model includes: Ant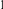, log percent sandy soil, and log percent chaparral (PMP = 30.8%). The top spatial regression model ranks 5th in order with a PMP of only 5.5% under the independence assumption. Table 4 shows the PIPs for each of the covariates. In addition, the table also shows the PIPs for the analysis without spatial correlation. When spatial correlation is included, log percent sandy soil is the only covariate with a PIP greater than 50%. When spatial correlation is ignored during the selection process Table 4 illustrates that a different model inference is obtained. Without a spatial model Ant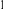 becomes a virtually certain inclusion and log percent chaparral also enters as an important variable along with log percent sandy soil.

**Table 3:** Model selection results for the California lizard data set.

Variables in model	Spatial PMP	Independent PMP (rank)
Ant 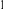 , log % Sandy soil	17.8	5.5 (5)
log % Sandy soil	13.9	★
Ant 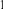 , log % Sandy soil, % Cover	11.7	★
log % Sandy soil, % Cover	10.1	★
Ant 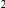 , log % Sandy soil	4.5	★

The top five models in PMP are shown. The PARJ chain visited 114 out of 128 possible models in the spatial analysis and 90 models were visited in the independence model. The table is ordered by PMP of the spatial regression analysis.

★ indicates PMP
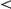
1.0 and Rank
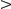
18.

**Table 4:** Posterior inclusion probabilities (PIP) for the California lizard data.

Environmental Covariate	Spatial PIP	Independent PIP
Ant 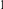	49.5	99.8
Ant 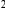	14.5	22.0
ln % Sandy Soil	88.4	75.3
Elevation	20.5	29.4
Bare Rock	6.1	10.7
% Cover	34.9	11.8
ln % Chaparral	7.0	76.6

The first column of probabilities are results from a spatial analysis with nugget and anisotropy parameters present in the model. The second column of probabilities resulted from an independence model.

The fact that log percent chaparral enters the model in the independence case but is absent in the spatial model could be due to a spurious regional effect. At nearby sites both the response and covariate are likely to have similar values due the spatial correlation of each variable. In the sample, high response values seem to be associated with high covariate values, but it is really the proximity of the sites to one another that is driving the relationship. The same is also no doubt true for the PIP increase in the Ant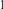 covariate.

The results obtained herein are similar to the previously mentioned analyses of this data. Using an isotropic Matern model with nugget [5], noted that log percent sandy soil seemed to be the most important covariate. Hoeting et al. [4] noted that the highest AIC model contained Ant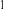 and log % sandy soil. While the MCMC analysis did not pick the full model under the independence assumption as AIC does [4], certainly more weight was placed on the other covariates. Using a spatial stepwise selection [3] also noted that the best model was one that contained ant abundance and log % sandy soil.

**Figure 1:**
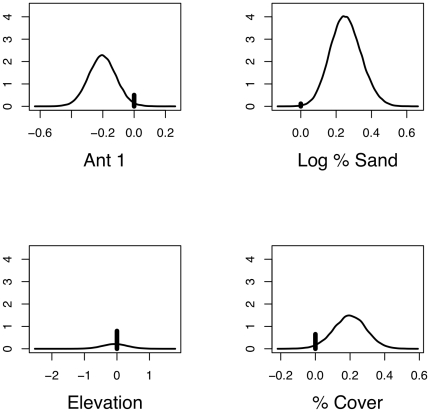
Marginal posterior density estimates for the top 4 PIP regression coefficients in the lizard abundance analysis. Included are coefficients are: Ant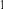 (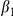), log % sand (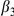), Elevation (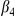), and % cover (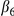). Vertical bars represent 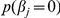 and the density curve is a kernel estimate conditioned on 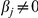.

In addition to the model inference obtained through the PARJ method, Figure 1 shows the marginal posterior density estimates for the top four PIP coefficients. Using these distributions direction and magnitude of the covariate effects can be examined after accounting for model uncertainty. Relative to high ant abundance, the presence of low ant abundance negatively influences lizard abundance. This should be expected as the ants are the main prey source. Lizard abundance is positively related to the percent of sandy soil in the substrate. The remaining coefficients, elevation and percent cover, have smaller PIP as can be seen in Figure 1 by the size of the bar relative to the density curve. Elevation seems to have neither strong positive or negative influence as the density curve is centered at zero. There seems to be a positive influence of percent cover, but there is substantial probability (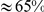) that it is equal to zero.

### Example 2: Abundance of Pollution Tolerant Fish

During the analysis, the PARJ chain visited 419 out of 512 possible models. The top five models, as determined by PMP, are given in Table 5. The results indicate that there is considerable uncertainty; the maximum *a posteriori* model accounts for only 12.4% of the total probability mass in the informative model and 17.8% of the mass in the flat prior analysis. The top PMP model coincides in each analysis. The models containing the predictors WSA and ELEV in the informative prior analysis ranked lower and had a smaller PMP than in the flat prior analysis. This suggests that WSA and ELEV are not as important to intolerant fish abundance as was described by the informative prior. This also explains the increase in maximum PMP. The data contradict the informative prior which leads to an increase in model uncertainty. Three covariates stand out as having significant probability (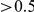) of inclusion in the regression model, stream order (ORDER), percentage of watershed disturbed by human use (DISTOT), and habitat quality (HAB) (Table 6). The same is true of the flat prior analysis. The PIPs are much smaller for ELEV and WSA than the informative model prior, leading to the conclusion that they are not as important to intolerant fish abundance *a posteriori*. All other covariates remain relatively unchanged with respect to PIP.

**Table 5:** Model selection results for the fish tolerance data set.

Variables in model	Informative PMP	Flat PMP (rank)
ORDER, DISTOT, HAB	12.4	17.8 ( 1)
ORDER, WSA, DISTOT, HAB	5.9	2.9 ( 6)
ORDER, RD, HAB,	5.4	8.9 ( 2)
ORDER, ELEV, DISTOT, HAB	4.8	2.4 (10)
ORDER, RD, DISTOT, HAB	3.8	5.5 ( 3)

Listed are the explanatory covariates selected using the PMP criterion. The table is ordered according to the PMPs of the informative prior analysis.

**Table 6:** Posterior inclusion probabilities (PIP) for the MAHA pollution intolerant fish data.

Environmental covariate	Informative PIP	Flat PIP
ORDER	88.3	84.1
ELEV	29.1	12.4
WSA	42.6	28.2
RD	38.0	40.1
DISTOT	78.7	76.3
HAB	73.8	74.4
DO	14.8	14.1
XFC	10.1	10.4
PCT	13.2	13.6

The first column gives PIPs for the informative model prior analysis. The second column gives PIPs for the flat model prior analysis.

**Figure 2:**
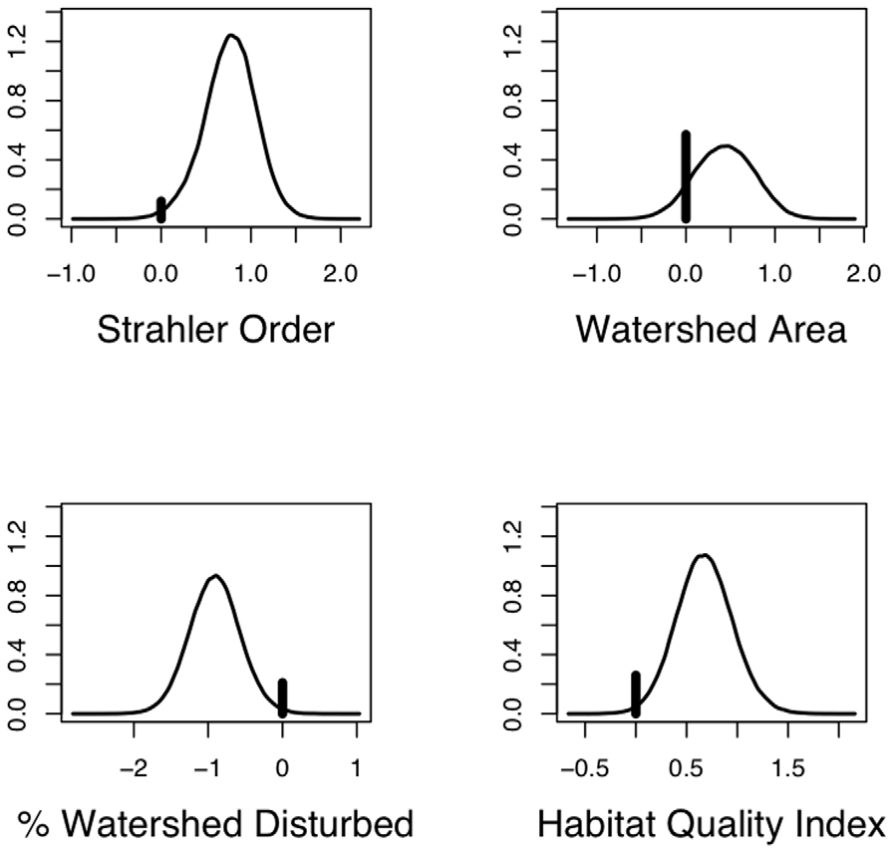
Marginal posterior density estimates for the top four PIP regression coefficients in the fish abundance analysis. Included are coefficients are: Strahler order (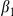), Watershed area (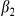), % watershed area disturbed by human use (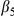), and habitat quality (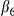). Vertical bars represent 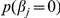 and the density curve is a kernel estimate conditioned on 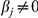.

Figure 2 illustrates the estimated marginal posterior densities for the four parameters: ORDER, WSA, DISTOT, and HAB. ORDER is positively related to intolerant fish abundance. WSA is also positively related to intolerant fish abundance, however, there is not strong evidence of a significant effect. Abundance is negatively related to DISTOT and positively related to HAB. Investigation of the scale of coefficient values for DISTOT and HAB shows that DISTOT seems to have a larger magnitude effect than HAB, suggesting a larger effect of watershed scale disturbance over site level effects. In fact, the PARJ output can be used to determine the model averaged posterior distribution of the ratio of the coefficients for DISTOT to HAB. The posterior probability that DISTOT has a larger magnitude coefficient is 60.4%, providing some evidence of a larger watershed level effect. When both variables are included in the model, however, the 95% highest posterior probability interval for the ratio of absolute coefficient values is 0.00–4.93, indicating the evidence is not strong.

## Discussion

To date, Bayesian methods of model selection and multimodel inference (i.e. model averaging) have not been utilized in great numbers of published research endeavors. No doubt this is due to the fact that PMPs are usually never available in closed form for many of the models typically used in ecology. This is also true, however, for information criterion such as AIC when generalized linear mixed models are utilized.

The Partial Analytic RJMCMC presented here should be considered a worthy competitor for information criterion methods such as AIC for spatial regression models. First, there is a practical implementation argument. The PARJ algorithm provides a consistent inference methodology for spatial regression modeling. This same method can be used for normal distribution models as well as spatial GLMMs for non-normal data such as counts. While an “off-the-shelf” RJMCMC procedure is often challenging to implement, the PARJ version is not any more difficult to program than a standard Gibbs MCMC sampler for a spatial regression model. One needs only add a model-jumping step. The analyses presented here were programmed in R where run times were on the order of a couple hours for the fish data. The second benefit of a PARJ is that it is Bayesian in nature. The PARJ approach allows information that the researcher possesses about the covariates to enter into the inference. The covariates can be weighted differentially *a priori*.

The PARJ method presented here is not limited to geostatistical regression models. Any covariance model can be used for the the latent process variable 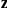. The PARJ method can be easily modified to make model inference with time series correlation or correlation due to factor random effects. This fact makes this method even more appealing as a general procedure for correlated data regression modeling.

## Supporting Information

File S1 **Reversible-jump MCMC details.** Additional material for implementing the RJMCMC methods presented in the paper.
(PDF)

File S2 **Convergence assessment.** Additional material on assessing convergence of the RJMCMC chains to their stationary distributions.
(PDF)

File S3 **Analysis code R.** R code for performing the analysis presented in the paper.
(R) Click here for additional data file.
