# Supplementary material for: Bayesian Multimodel Inference for Geostatistical Regression Models
Source: PLoS One. 2011 Nov 10;6(11):e25677. doi: 10.1371/journal.pone.0025677 (PMC3213087; doi:10.1371/journal.pone.0025677)
Supplement: File S1 — Reversible-jump MCMC details. Additional material for implementing the RJMCMC methods presented in the paper. (PDF) [file pone.0025677.s001.pdf]

Supplementary Information S1: Reversible Jump Markov chain Monte Carlo Procedure

Within Model Moves

This section describes the details for making parameter updates for within model moves. These steps are necessary even if parameter value inference is not the main objective. This must be done in order to maintain the correct properties of the Markov transitions for the MCMC. Updating the parameters in step 1 proceeds as follows:

1. update θ_1, θ_2, α_1, and α_2 separately with the following generic procedure:
   (a) Let ϑ be the current value of one of the spatial parameters.
   (b) Draw a proposal ϑ^* from $N(\vartheta^*|\vartheta, \gamma)$, where γ is a tuning parameter set by the researcher to promote good mixing (usually done by trial and error).
   (c) Calculate
   $$R(\vartheta^*; \vartheta) = \frac{N(\mathbf{z}|\mathbf{X}_k\boldsymbol{\beta}_k, \boldsymbol{\Sigma}^*)p(\vartheta^*)}{N(\mathbf{z}|\mathbf{X}_k\boldsymbol{\beta}_k, \boldsymbol{\Sigma})p(\vartheta)}$$
   (d) Draw U from a Uniform(0,1) distribution. If $U < R(\vartheta^*; \vartheta)$, set ϑ^* as the new value, else retain the old value as the current value.

2. Update the anisotropy parameter ψ in the same general way as the other spatial parameters.
   (a) Draw ψ^* from a uniform distribution with lower bound $\kappa(\psi) = \max\{-1, \psi - \eta\}$ and upper bound $\lambda(\psi) = \min\{1, \psi + \eta\}$, where η is a tuning parameter.
   (b) Draw $U \sim U(0, 1)$ and accept ψ^* if U is less than
   $$R(\psi^*; \psi) = \frac{N(\mathbf{z}|\mathbf{X}_k\boldsymbol{\beta}_k, \boldsymbol{\Sigma}^*)p(\psi^*)}{U(\psi^*|\kappa(\psi), \lambda(\psi))} \times \frac{U(\psi|\kappa(\psi^*), \lambda(\psi^*))}{N(\mathbf{z}|\mathbf{X}\boldsymbol{\beta}_k, \boldsymbol{\Sigma})p(\psi)}$$

3. Draw new $\boldsymbol{\beta}_k$ value from full conditional distribution
   $$p(\boldsymbol{\beta}^*| \dots) = N(\boldsymbol{\beta}_k^*|\hat{\boldsymbol{\mu}}_k, \widehat{\mathbf{V}}_k)$$
   where $\widehat{\mathbf{V}}_k = \mathbf{V}_k^{-1} + \mathbf{X}_k'\boldsymbol{\Sigma}^{-1}\mathbf{X}_k$ and $\hat{\boldsymbol{\mu}}_k = \widehat{\mathbf{V}}_k\left(\mathbf{V}_k^{-1}\boldsymbol{\mu}_k + \mathbf{X}_k'\boldsymbol{\Sigma}^{-1}\mathbf{z}\right)$. No Metropolis-Hastings acceptance evaluations are necessary since the full conditional distribution is a common form and updates can be easily drawn.

4. Update $\mathbf{z}$ with a Langevin-Hastings proposal [1].

(a) Let $\mathbf{z}$ be the current value of the latent spatial process

(b) Draw candidate update $\mathbf{z}^*$ from a normal distribution with mean $\zeta(\mathbf{z}) = \mathbf{z} + \frac{h}{2}\nabla \log\left\{p(\mathbf{y}|\mathbf{z})N(\mathbf{z}|\mathbf{X}\boldsymbol{\beta}_k, \boldsymbol{\Sigma})\right\}$ and covariance matrix $h\mathbf{I}_n$, where ∇ represents the derivative with respect to $\mathbf{z}$. For the fish abundance example

$$\zeta(\mathbf{z}) = \mathbf{y} - \exp\{\mathbf{z}\} - \boldsymbol{\Sigma}^{-1}(\mathbf{z} - \mathbf{X}_k\boldsymbol{\beta}_k);$$

heuristically a very sensible choice for drift, the term contrasts likelihood fit versus spatial spatial smoothing.

(c) As with the spatial parameters, draw a standard uniform variable U. If U is less than

$$R(\mathbf{z}^*; \mathbf{z}) = \frac{p(\mathbf{y}|\mathbf{z}^*)N(\mathbf{z}^*|\mathbf{X}_k\boldsymbol{\beta}_k, \boldsymbol{\Sigma})\exp\left(-\|\mathbf{z} - \zeta(\mathbf{z}^*)\|^2/(2h)\right)}{p(\mathbf{y}|\mathbf{z})N(\mathbf{z}|\mathbf{X}_k\boldsymbol{\beta}_k, \boldsymbol{\Sigma})\exp\left(-\|\mathbf{z}^* - \zeta(\mathbf{z})\|^2/(2h)\right)},$$

accept the new proposal, else retain the current value.

General Reversible-Jump and PARJ MCMC

The general RJMCMC method proceeds as follows for a current state $q = (\boldsymbol{\vartheta}_k, m_k)$:

1. Draw proposal move of type i to m_{k^*} from distribution $J_i(q)$
2. Draw parameter proposal $\boldsymbol{\vartheta}_{k^*}$ from $G_i(q, m_{k^*})$
3. Accept new state q^* with probability

$$\min\left\{1, \frac{p(q^*|Data)J_i(q^*)G_i(q^*, m_k)}{p(q|Data)J_i(q)G_i(q, m_{k^*})}\right\}. \tag{1}$$

Typically, an RJMCMC algorithm involves several move types in order to obtain an ergodic chain. Move types can be systematically or randomly selected. Both Metropolis-Hastings and Gibbs samplers are special cases of RJMCMC [2]. The PARJ acceptance ratio for spatial regression models results from substitution of $G_i(x) = p(\boldsymbol{\beta}_{k^*}|m_{k^*}, \boldsymbol{\xi}, \mathbf{z})$ in (1) and the identity

$$p(m_{k^*}|\boldsymbol{\xi}, \mathbf{z}) = \frac{p(\boldsymbol{\beta}_k, m_{k^*}|\boldsymbol{\xi}, \mathbf{z})}{p(\boldsymbol{\beta}_{k^*}|m_{k^*}, \boldsymbol{\xi}, \mathbf{z})}.$$

To obtain the acceptance probability ratio note that if $p(\boldsymbol{\beta}_k|\boldsymbol{\xi}) = N(\boldsymbol{\mu}_k, \mathbf{V}_k)$, then, since $\mathbf{z} = \mathbf{X}_k\boldsymbol{\beta}_k + \boldsymbol{\delta}$, one obtains $p(\mathbf{z}|\boldsymbol{\xi}, m_k) = N(\mathbf{X}_k\boldsymbol{\mu}_k,\ \mathbf{X}_k\mathbf{V}_k\mathbf{X}_k' + \boldsymbol{\Sigma})$, where $\boldsymbol{\Sigma} = Cov(\boldsymbol{\delta})$. Finally, Bayes theorem is used

to obtain

$$
\begin{aligned}
p(m_k|\boldsymbol{\xi}, \mathbf{z}) &= \frac{p(\mathbf{z}|\boldsymbol{\xi}, m_k)p(m_k|\boldsymbol{\xi})}{p(\mathbf{z}|\boldsymbol{\xi})} \\
&\propto p(\mathbf{z}|\boldsymbol{\xi}, m_k)p(m_k) \\
&\propto \exp\left\{-\frac{1}{2}(\mathbf{z} - \mathbf{X}_k\boldsymbol{\mu}_k)'(\mathbf{X}_k\mathbf{V}_k\mathbf{X}_k' + \boldsymbol{\Sigma})^{-1}(\mathbf{z} - \mathbf{X}_k\boldsymbol{\mu}_k)\right\} p(m_k).
\end{aligned} \tag{2}
$$

The ratio of the previous calculation evaluated at the proposed model m_{k^*} to the current model m_k forms the acceptance probability for model jump. Only the model proposal is needed to calculate the ratio.

References

1. Christensen OF, Waagepetersen R (2002) Bayesian prediction of spatial count data using generalized linear mixed models. Biometrics 58: 280–286.

2. Green PJ (2003) Trans-dimensional markov chain monte carlo. In: Green PJ, Hjort NL, Richardson S, editors, Highly Structured Stochastic Systems, New York: Oxford University Press, Inc.
