# Supplementary material for: Bayesian Multimodel Inference for Geostatistical Regression Models
Source: PLoS One. 2011 Nov 10;6(11):e25677. doi: 10.1371/journal.pone.0025677 (PMC3213087; doi:10.1371/journal.pone.0025677)
Supplement: File S2 — Convergence assessment. Additional material on assessing convergence of the RJMCMC chains to their stationary distributions. (PDF) [file pone.0025677.s002.pdf]

Supplementary Information S2: Convergence Diagnostics

In this appendix we present an analysis of convergence for the examples presented in the main paper. Unfortunately, because the RJMCMC jumps between models and thus parameter dimension changes, there is no straightforward method to calculate a convergence diagnostic [1]. Specific parameters "come and go" throughout the course of the chain. The general consensus in various proposed methods [1] is to monitor common parameters in each of the models. So, with that in mind we assessed convergence of the common parameters β_0, $\ln \tau$, $\ln \sigma$, and $\boldsymbol{\alpha}$, and ψ using the Heidelberg and Welch [2] diagnostic (HW) tests. The HW procedure employs two tests. The first test determines whether the trace of simulated values arises from a stationary stochastic process. The second test determines if there are enough iterations to estimate the mean of the process with acceptable precision. The HW tests were chosen because it requires only one realization of the MCMC to use. Spatial models are often computationally burdensome so running multiple chains may be unrealistic for some analysis. We used the `R` package `coda` to implement the diagnostic via the function `heidle.diag()`. Default values were for all arguments of the function were used. In addition, each of the trace plots of the regression coefficients and model state are presented to examine any systematic deviation from the steady state of a converged MCMC chain as well as the autocorrelation plots for the common parameters. As one might expect, the chain for the parameters of the GLMM model for fish abundance had higher autocorrelation. Thus, the chain was run 3 times as long as the whiptail RJMCMC to reach satisfactory assurance that the chain was stationary.

Example 1: Analysis of Whiptail Lizard Abundance (Anisotropic Model)

Table 1. Heidelberger-Welch stationary test for the analysis of whiptail lizard abundance.

Parameter	Test	Iteration	p-value
β_0	passed	1	0.0816
$\ln\sigma$	passed	1	0.9167
$\ln\tau$	passed	1	0.6944
α_1	passed	1	0.3284
α_2	passed	1	0.4038
ψ	passed	1	0.5317

Table 2. Heidelberger-Welch halfwidth test for the analysis of whiptail lizard abundance.

Parameter	Test	Mean	Halfwidth
β_0	passed	-2.407	0.00686
$\ln\sigma$	passed	1.221	0.01209
$\ln\tau$	passed	-0.482	0.00260
α_1	passed	0.713	0.01501
α_2	passed	-0.325	0.01831
ψ	passed	0.109	0.00429

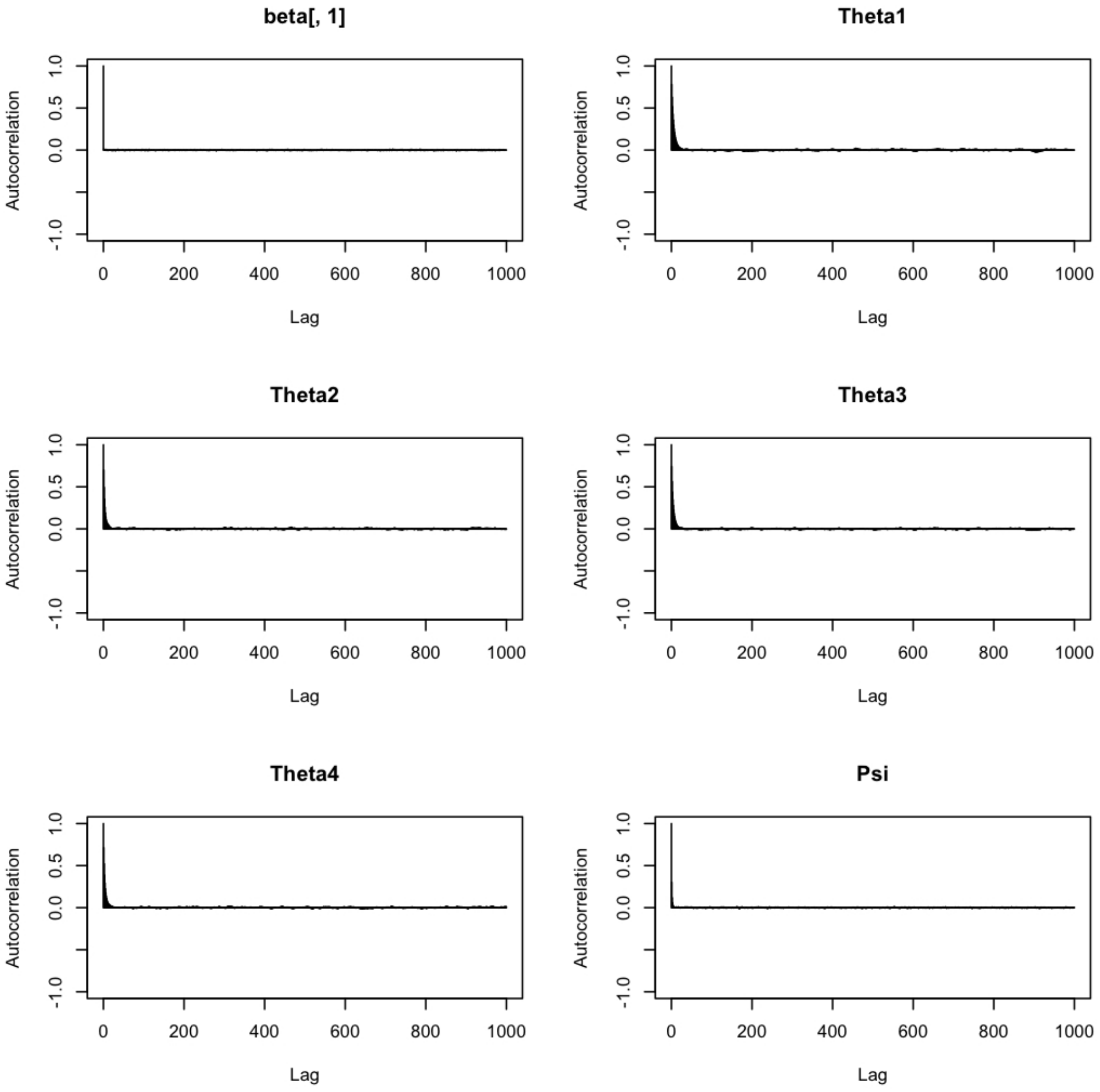

Figure 1. Autocorrelation function plot. Plot of autocorrelation values at various lags for the whiptail lizard RJMCMC chain.

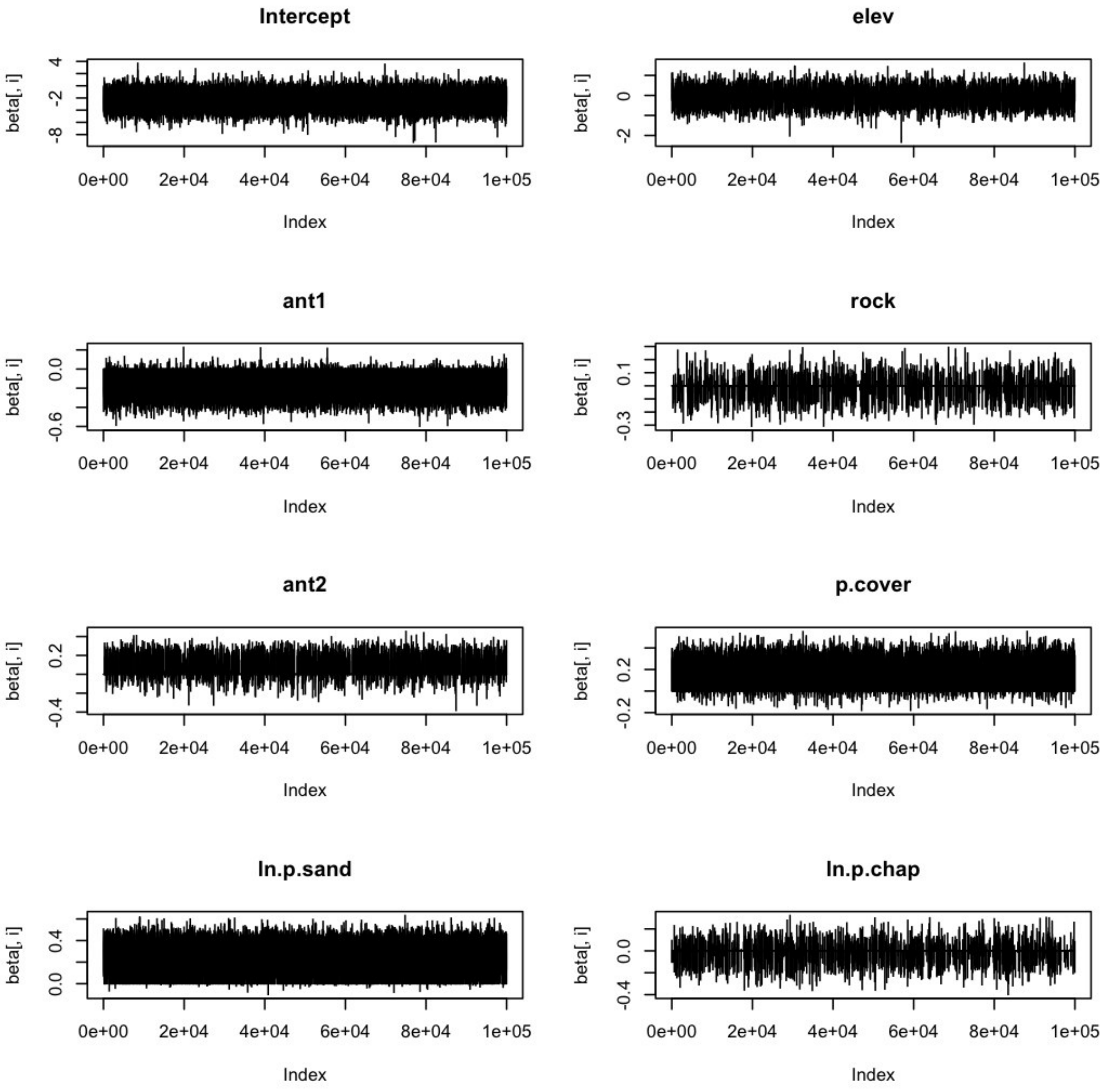

Figure 2. MCMC trace plots. Coefficient trace plots for the anisotropic spatial model of whiptail lizard abundance.

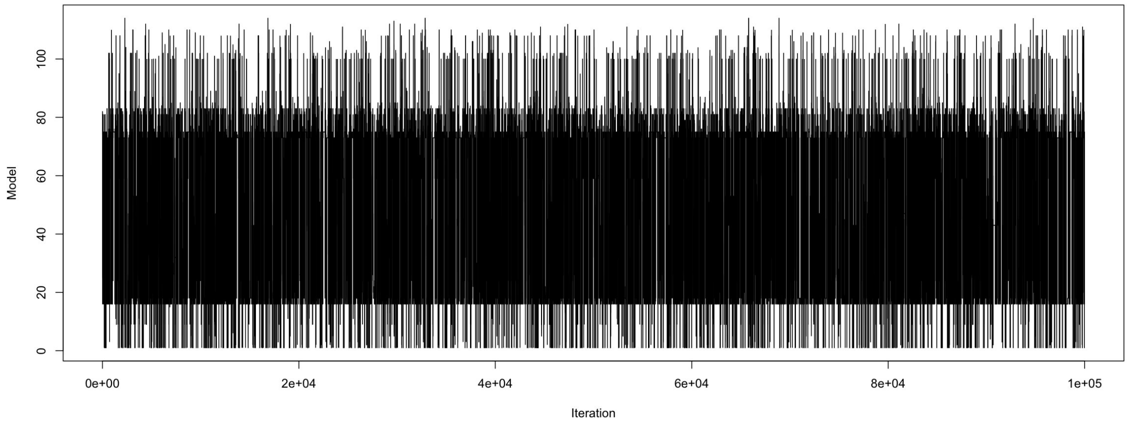

Figure 3. Trace plot of model stops. Here we present a trace plot of the categorical model chain for the whiptial lizard analysis. The models are numbered in the order that they are first visited.

Example 2: Abundance of Pollution Tolerant Fish (Flat Prior Model Probability Analysis)

Table 3. Heidelberger-Welch stationary test for the analysis of pollution intolerant fish abundance.

Parameter	Test	Iteration	p-value
β_0	passed	90001	0.0745
$\ln \sigma$	passed	1	0.0910
α	passed	30001	0.0644

Table 4. Heidelberger-Welch halfwidth test for the analysis of pollution intolerant fish abundance.

Parameter	Test	Mean	Halfwidth
β_0	passed	0.549	0.0382
$\ln \sigma$	passed	0.859	0.0152
α	passed	-2.782	0.0848

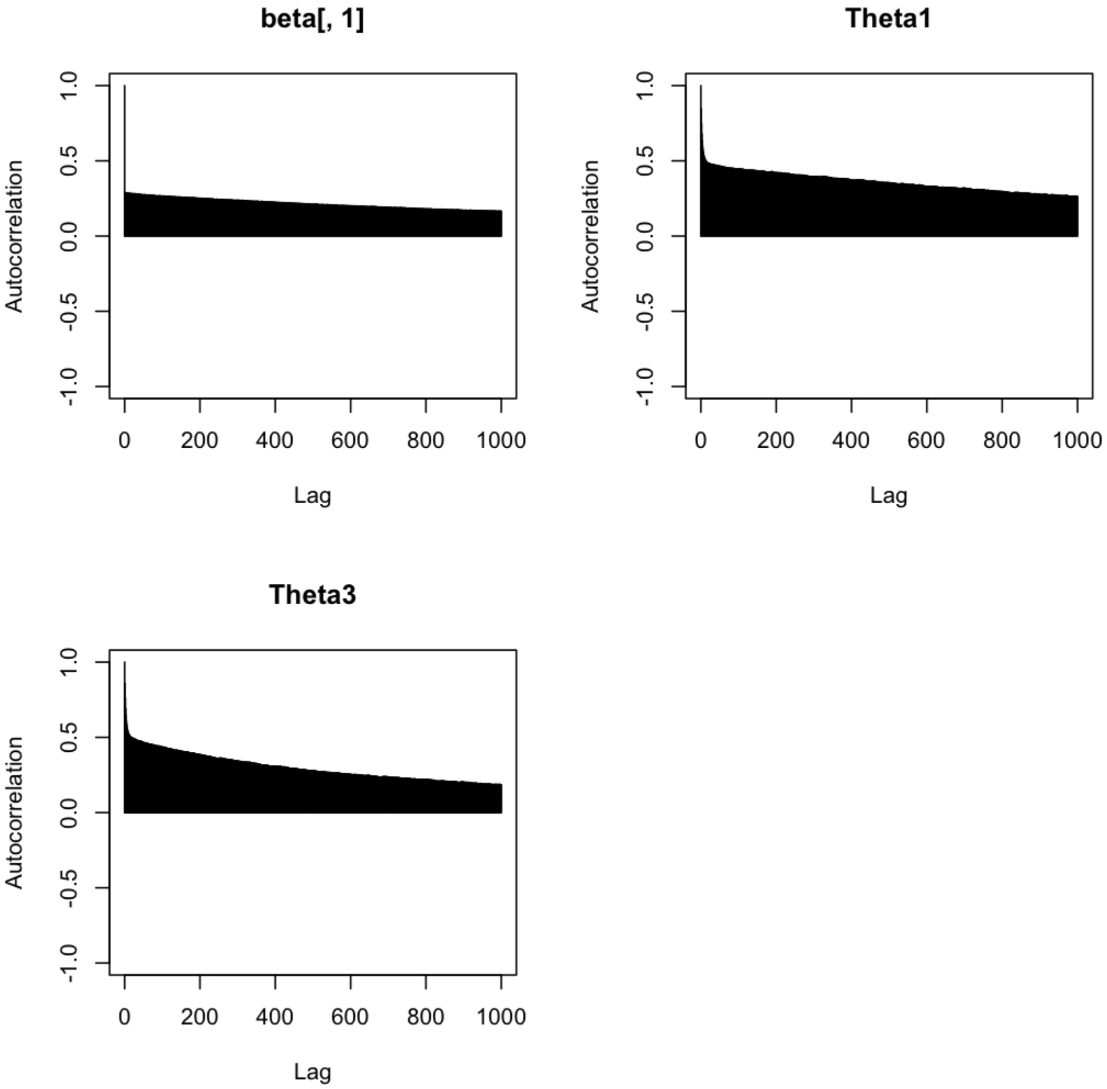

Figure 4. Autocorrelation function plot. Plot of autocorrelation values at various lags for the fish abundance RJMCMC chain.

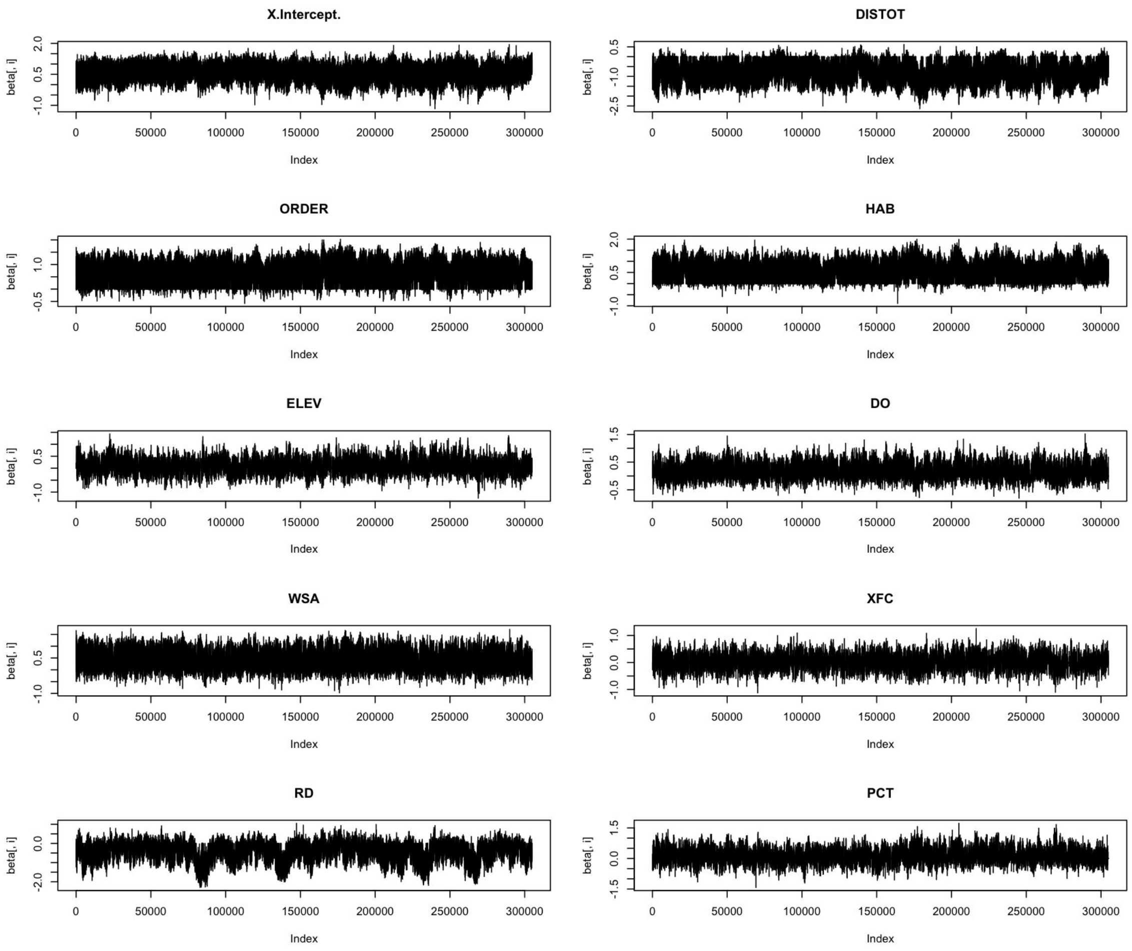

Figure 5. MCMC trace plots. Coefficient trace plots for the even model prior probabilities analysis of pollution intolerant fish abundance.

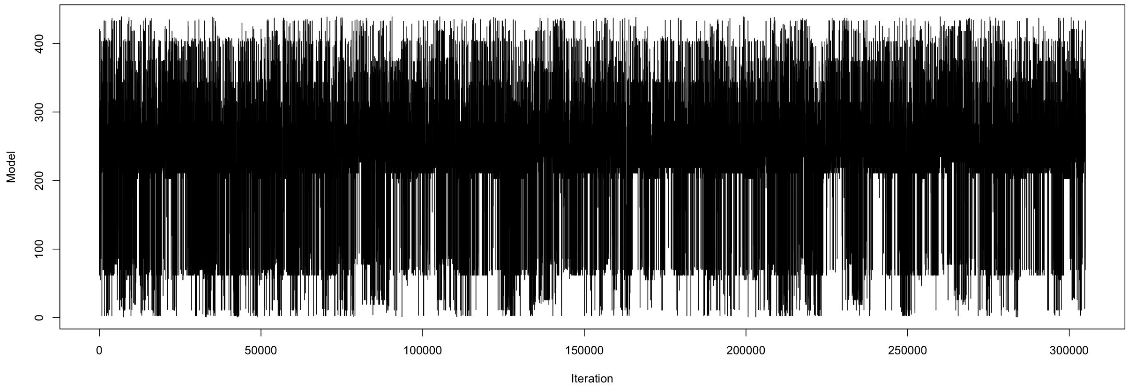

Figure 6. Trace plot of model stops. Here we present a trace plot of the categorical model chain for the fish abundance analysis. The models are numbered in the order that they are first visited.

References

1. Sisson S (2005) Transdimensional markov chains: A decade of progress and future perspectives. Journal of the American Statistical Association 100: 1077–1089.

2. Heidelberger P, Welch P (1983) Simulation run length control in the presence of an initial transient. Operations Research 31: 1109–1144.
